# Outcome and prognosis of secondary lung cancer surgery with interstitial lung disease

**DOI:** 10.1111/1759-7714.14481

**Published:** 2022-05-30

**Authors:** Hideomi Ichinokawa, Kazuya Takamochi, Mariko Fukui, Aritoshi Hattori, Takeshi Matsunaga, Kenji Suzuki

**Affiliations:** ^1^ Department of General Thoracic Surgery Juntendo University Hospital Tokyo Japan

**Keywords:** interstitial lung disease, lung cancer, postoperative complication, recurrence, second surgery

## Abstract

**Background:**

The initial surgery for lung cancer with interstitial lung disease (ILD) is often followed by passive treatment due to the surgery‐induced deterioration in respiratory function, and only a few studies have summarized the findings associated with a second surgery for lung cancer patients with ILD.

**Methods:**

Of the 3932 lung cancer patients who underwent surgery at our hospital from August 2008 to July 2019, 404 (10%) patients (1) underwent preoperative computed tomography for imaging of interstitial pneumonia and (2) underwent initial surgery. We analyzed 45 cases (11%) suspected of showing metachronous lung cancer during the postoperative course.

**Results:**

Thirty‐four patients (76%) underwent a second surgery. The group that underwent a second surgery showed a significantly better prognosis than the group that did not (*p* = 0.0009). The surgical procedure was wide‐wedge resection/segmentectomy/lobectomy and above in 15/7/12 cases, respectively. Postoperative complications were observed in nine cases (26%) (prolonged pulmonary fistula in five cases, ILD acute exacerbation in two cases, and wound dissection in two cases). Mortality within 30 days occurred in one case (ILD acute exacerbation at postoperative day 15). Twelve patients (35%) experienced recurrence. In the wide‐wedge resection group, 2/15 (13%) patients showed stump recurrence. The 1‐, 2‐, 3‐, and 5‐year survival rates after surgery for secondary lung cancer were 80.4, 72.5, 68.2, and 39.4%, respectively.

**Conclusion:**

Surgery can be considered an effective treatment method for secondary lung cancer with ILD if the cases are carefully selected.

## Graphical abstract

As a second treatment, the surgery group with interstitial lung disease (ILD) showed a significantly better prognosis than the nonsurgery group with ILD (*p* = 0.0092). Choosing limited surgery has an advantage of preservation of respiratory function, but a disadvantage of stump recurrence (2/15, 13%). On the other hand, choosing standard surgery (lobectomy and more than lobectomy) has an advantage of no stump recurrence, but has a disadvantage of decreased respiratory function.

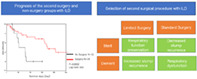

## INTRODUCTION

The frequency of detection of metachronous multiple lung cancers has recently increased,^1^, ^2^ and one reason for this increase is the progress and spread of diagnostic imaging modalities such as chest computed tomography (CT) examination, which has increased the chances of finding multiple lung nodules. In particular, patients with interstitial lung disease (ILD) are more likely to develop lung cancer than those without ILD.^3^ Multiple metastatic lung cancers have a better prognosis after surgery than intralobar metastatic or recurrent lung cancer.^4^ However, the surgical procedure in ILD patients is more restricted than that in other patients, especially in relation to the treatment of the first cancer, the site and degree of progression of the second cancer, and respiratory function. Thus, formulation of a treatment strategy is expected to be difficult. Moreover, acute exacerbation (AE) of ILD and a decline in respiratory function between the first and second operations is also highly possible. Nevertheless, only one study has summarized the findings associated with a second surgery for lung cancer patients with ILD.^5^ Here, we report the results and prognosis of a second surgery for patients with ILD‐complicated lung cancer.

## METHODS

### Study population

**FIGURE 1:**
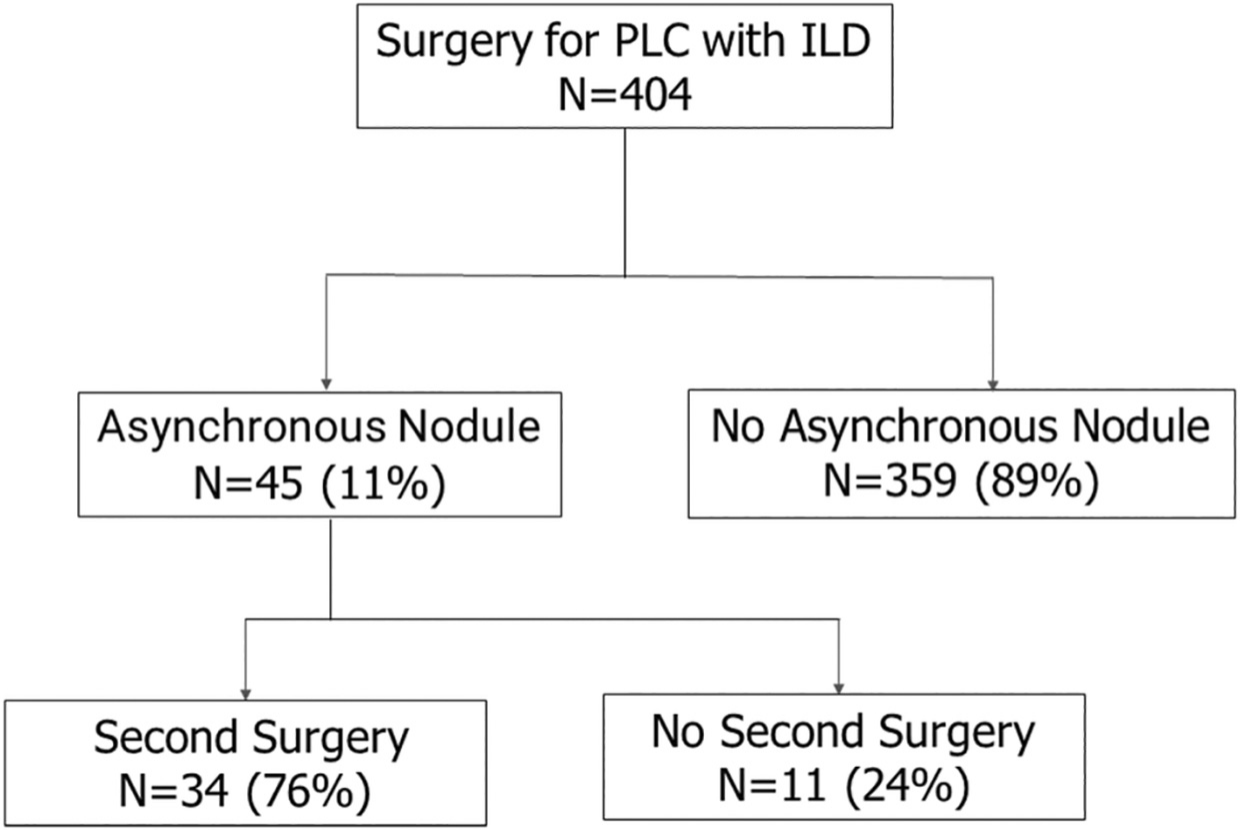
Patient distribution in this study

This retrospective study was approved by the ethics committee of our institution (20210309). Between August 2008 and July 2019, surgeries were performed in 3932 cases of pulmonary lung cancer at our institution. Among them, 404 patients (10%) who showed ILD on a chest CT and underwent the first operation were examined. The first surgery revealed 198 postoperative complications (49.0%). A total of 17 cases (4.2%) of AE occurred within 30 days of the operation. Five (1.2%) and 21 cases (5.2) died within 30 and 90 days of the operation, respectively. These 404 cases included 45 cases (11%) showing metachronous nodules after the first surgery (including two cases of stump recurrence). We then divided the 45 patients into two groups: those who underwent a second surgery (group A, *n* = 34) and those who did not (group B, *n* = 11) (Figure 1). The reasons for not performing surgery in group B were as follows: patient refusal, four cases; lack of surgical function, seven cases. We analyzed the following clinical background characteristics as well as the peri‐ and postoperative results: age at first surgery, sex, preoperative comorbidities, smoking history, clinical staging, Japanese Association for Chest Surgery (JACS) risk score, respiratory function (vital capacity [VC], %VC, forced expiratory volume in one second [FEV1.0], FEV1.0%, % predicted FEV1.0 (%FEV1.0), percentage diffusing capacity for carbon monoxide [%DL_CO_]), first and second surgical procedures, mediastinal lymph node dissection, operation time, intraoperative blood loss, introduction of home oxygen therapy, histopathological findings, pathological stage, hospital stay, Clavien–Dindo grade ≥2 postoperative complications, presence or absence of recurrence, and recurrence treatment method. We also evaluated the surgical morbidity and mortality rates within 30 and 90 days of the operation. We defined “wide‐wedge resection and segmentectomy” as “limited surgery”. The median observation period from the first surgery was 1619 days.

### Definition of metachronous lung cancer

We performed a comprehensive diagnosis of metachronous multiple lung cancer by referring to the diagnostic criteria proposed by Warren et al.^6^ and Martini et al.,^7^ and the diagnostic criteria of the American College of Chest Physicians presented by Shen et al.^8^ In cases with no evidence of a histological diagnosis, the patient was clinically diagnosed with metachronous lung cancer on the basis of the following guidelines: (1) The radiologist diagnosed the primary lung cancer as suspicious based on the imaging findings. (2) A nodule showed a gradual growth tendency for more than 6 months after the clinical course. (3) No lesions suspected to be malignant other than lung lesions were found on whole‐body CT or positron emission tomography (PET)‐CT examinations. (4) New nodules appearing after surgery for the first lung cancer were visualized as an isolated shadow without lymph node swelling. However, the two cases of stump recurrence described above were not included.

### Diagnosis of ILD

All patients underwent preoperative thoracic computed axial tomography with a slice thickness of ≤3 mm and mediastinal (level, 40 Hounsfield units [HU]; width, 400 HU) and lung (level, 600 HU; width, 1600 HU) window settings to evaluate the primary tumor and mediastinal nodes. All CT findings were reviewed anew by the authors (two radiologists, Kazuhiro Suzuki and Akihiro Hotta; one pulmonary medicine physician, Kazuhiro Ando; and two thoracic surgeons, Mariko Fukui and Kazuya Takamochi). Published criteria were used to categorize CT findings as usual interstitial pneumonia (UIP), probable UIP, indeterminate for UIP, and alternative diagnosis.^9^ Our patients did not include those with connective tissue and autoimmune diseases. Cases with UIP and probable UIP patterns were considered UIP pattern‐positive.

### Japanese Association for Chest Surgery risk score

Patient background characteristics were compared not only using the raw data extracted from medical charts, but also by the JACS risk score, which evaluated the history of AE (5 points), anatomic resection more extensive than segmentectomy (4 points), UIP pattern (4 points), preoperative steroid usage (3 points), male sex (3 points), KL‐6 level >1000 U/ml (2 points), and %VC <80% (1 point).^10^

### Statistical analysis

Descriptive statistics were used to assess the patients' demographic characteristics and outcomes. Normally distributed continuous data were expressed as median values, and categorical data were expressed as counts and proportions. Survival was calculated using the Kaplan–Meier method, and differences in survival were assessed using log‐rank analysis. Comparisons among all parameters were analyzed using the Student's *t‐*test. We performed multivariate analysis of late AEs by using the Cox proportional hazards regression model, and used a logistic regression model of the results of the multivariate analysis to identify the risk factors for late AE. All data were analyzed using SPSS software (version 23.0; SPSS Inc.). Statistical significance was set at *p* < 0.05.

## RESULTS

### Comparison of preoperative clinical features between surgery and nonsurgery groups (group A vs. group B)

Table 1 shows the preoperative clinical features of groups A and B. All patients in group B (11 cases) were male. Patients in group A showed significantly better respiratory function (VC, %VC, FEV1.0, FEV1.0%, %FEV1.0, %DL_CO_) than those in group B (*p* < 0.05). The two groups showed no significant differences in age at first surgery, sex, preoperative comorbidities, pack‐year smoking, initial clinical stage, and JACS risk score.

**TABLE 1:** Clinicopathological comparison between the groups with and without second surgery

Variables	Group A (surgery) (*n* = 34)	Group B (no surgery) (*n* = 11)	*p*‐value
Age at first surgery [IQR]	72 [68–76]	71 [66–80]	0.51
Male sex	28 (82%)	11 (100%)	0.13
Preoperative comorbidity (without interstitial lung diseases)	28 (82%)	10 (91%)	0.50
Smoking pack‐years [IQR]	46.5 [36–60]	60.0 [46–100]	0.053
Initial pathological stage (I/II/III)	20/10/4	5/5/1	0.44
JACS risk score [IQR]	7 [7–11]	11 [7–11]	0.14
VC, L [IQR]	3.40 [2.80–4.06]	2.86 [2.14–3.07]	0.022
%VC, % [IQR]	104.1 [92.3–119.1]	80.6 [71.1–91.5]	0.004
FEV1.0, L [IQR]	2.26 [1.98–2.71]	1.43 [1.10–1.80]	0.001
FEV1.0%, % [IQR]	69.7 [64.8–77.4]	63.0 [42.4–72.2]	0.008
%FEV1.0, % [IQR]	93.4 [83.0–102.3]	75.2 [58.8–104.5]	0.001
%DLCO, % [IQR]	47.0 [39.2–58.5]	32.4 [27.6–44.0]	0.010

Abbreviations: DLCO, diffusing capacity for carbon monoxide; FEV1.0, forced expiratory volume in 1 s; IQR, interquartile range; JACS, Japanese Association for Chest Surgery; VC, vital capacity.

### Details and prognosis of the nonsurgery group (group B)

Subsequent treatment for patients who did not undergo surgery (group B) included chemotherapy in three patients, stereotactic radiation therapy in two patients, and best supportive care in six patients. In group B, the survival rates at 1, 2, and 3 years were 60.0, 20.0, and 20.0%, respectively. Eight patients died, of which seven died from lung cancer and one died from other diseases.

### Details of the second surgery in the surgery group (group A)

Table 2 provides information regarding the second surgery in group A. Lobectomy was performed as the first surgical procedure in 26 cases (76%) and as the second surgical procedure in 12 cases (35%). Ipsilateral surgery was performed in 11 cases (32%): on the right side in eight cases (24%) and the left side in three cases (9%). Lymph node dissection was performed in 10 cases (29%). The most common postoperative complication was prolonged pulmonary fistula (5 cases), wherein ipsilateral surgery was performed on the right side. The number of intrathoracic administrations of OK‐432 for pulmonary fistula closure was 1/2/3/4 times in one, two, one, and one patients, respectively. Two cases showed ILD‐AE. The first case involved a 68‐year‐old man. He underwent left upper lobectomy, developed ILD‐AE at postoperative day (POD) 7, and underwent steroid pulse therapy (methylprednisolone 1000 mg) for 3 days. Steroid pulse therapy (methylprednisolone 1000 mg) was readministered at POD 12, but the patient died at POD 14 without any improvement in ILD‐AE. The second case involved a 66‐year‐old man. He underwent right lower lobectomy and showed ILD‐AE at POD 2. He received steroid pulse therapy (methylprednisolone 1000 mg) for 3 days and was ventilated for 3 POD. He was extubated at POD 8, introduced to home oxygen therapy (HOT) at POD 30, and was discharged. HOT was introduced in seven cases. In the second surgery, the surgical procedure was lobectomy in five cases and wide‐wedge resection in two cases. HOT became unnecessary within 3 months after the second operation in three cases. Among the four cases in which HOT was continued for 4 months or more, three involved lobectomy as the second surgical procedure. Only one patient died within 30 days, and the death was attributable to ILD‐AE.

**TABLE 2:** Intraoperative features of the second surgical group (group A)

Variables	Group A (surgery) (*n* = 34)
First surgical procedure	
Wide‐wedge resection	4 (12%)
Segmentectomy	4 (12%)
Lobectomy and more than lobectomy	26 (76%)
Surgical site	
Same side Right → Right	8 (24%)
Left → Left	3 (9%)
The other side Right → Left	15 (44%)
Left → Right	8 (24%)
Second surgical procedure	
Wide‐wedge resection	15 (44%)
Segmentectomy	7 (21%)
Lobectomy and more than lobectomy	12 (35%)
Mediastinal lymph node dissection	10 (29%)
Length of operation, minutes [IQR]	121 [82–166]
Blood loss, ml [IQR]	10 [5–25]
Hospital stay, days [IQR]	8 [7–13]
Postoperative complications	9 (26%)
Prolonged pulmonary fistula	5 (14%)
Acute exacerbation of interstitial lung disease	2 (6%)
Wound infection	2 (6%)
Home oxygen therapy	7 (21%)
30‐day mortality	1 (3%)
90‐day mortality	0 (0%)

Abbreviation: IQR, interquartile range.

### Prognosis in the surgery group (group A) (comparison of prognosis with nonsurgery group [group B])

**FIGURE 2:**
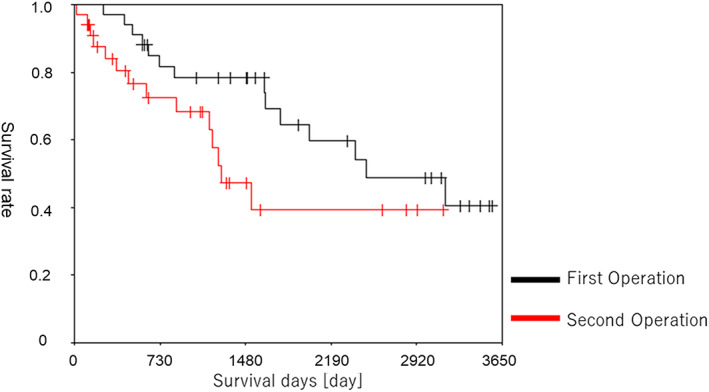
Prognosis after the first and second surgeries (group A)

**FIGURE 3:**
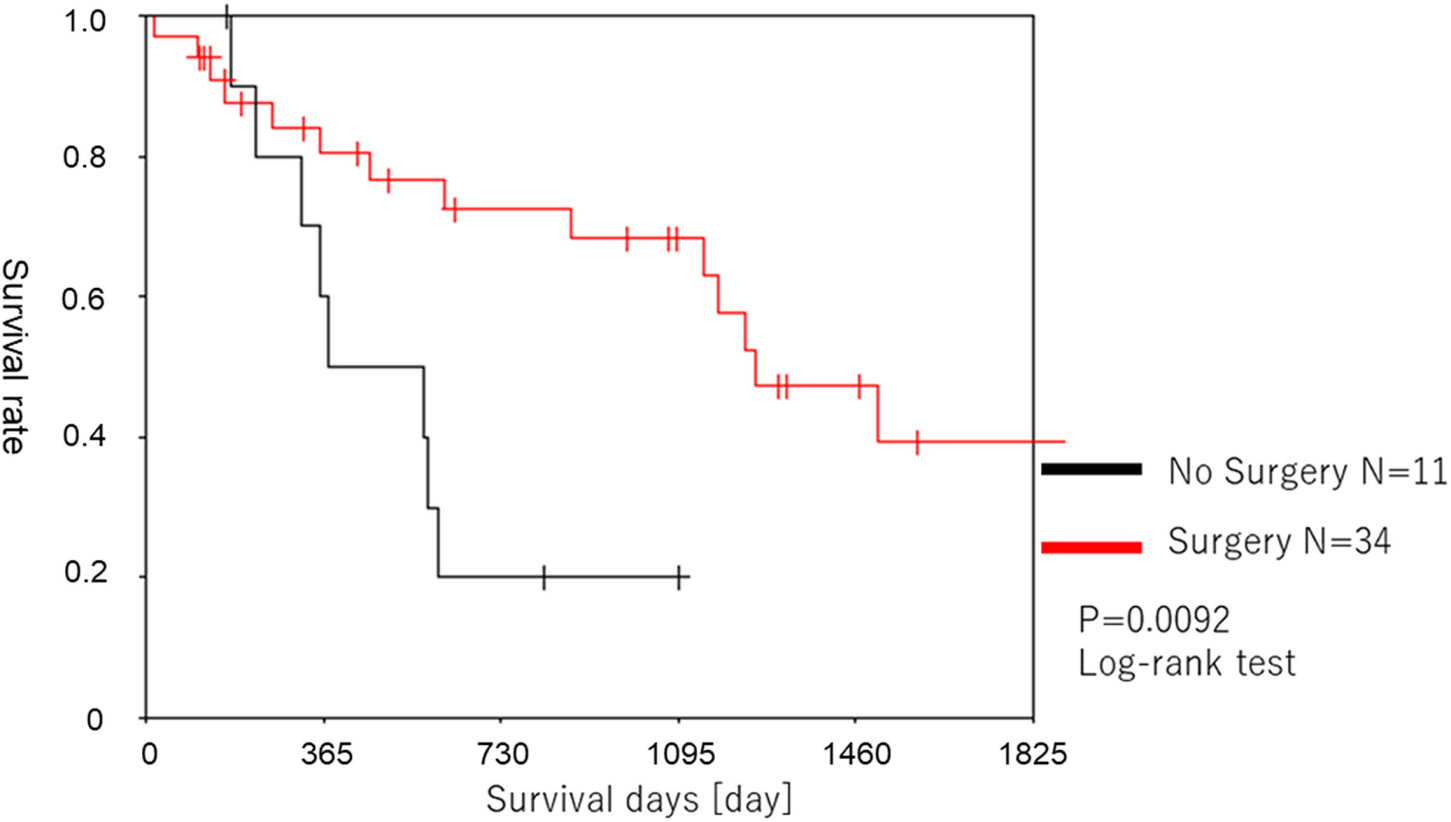
Prognosis of the second surgery (group A) and nonsurgery groups (group B)

Figure 2 shows the prognosis of the patients in group A. The 2‐, 3‐, 5‐, and 10‐year survival rates from the first surgery were 81.7, 78.4, 64.6, and 40.7%, respectively. The 1‐, 2‐, 3‐, and 5‐year survival rates after surgery for secondary lung cancer were 80.4, 72.5, 68.2, and 39.4%, respectively. Figure 3 provides a comparison of the prognoses of groups A and B after the second treatment. The surgery group (group A) showed a significantly better prognosis than the nonsurgery group (group B) (*p* = 0.0092). Table 3 describes the postoperative course in group A. The pathological findings were adenocarcinoma in 19 cases, squamous cell carcinoma in 12 cases, and others in three cases. Stage I, II, and III disease was identified in the pathological assessments in 26, four, and two cases, respectively. Recurrence after the second surgery was observed in 12 (35%) patients, including 11 cases of local recurrence and one case of distant metastasis. Treatment after recurrence involved surgery in two cases, radiation therapy in four cases, and best supportive care in six cases.

**TABLE 3:** Postoperative features of the second surgical group (group A)

Variables	Group A (surgery) (*n* = 34)
Pathology (Ad/Sq/Others)	19/12/3
Pathological stage (I/II/III/stump recurrence)	26/4/2/2
Recurrence case	12 (35%)
Local metastasis	11 (32%)
Distant metastasis	1 (3%)
Recurrence treatment	
Surgery	2 (17%)
Radiotherapy	4 (33%)
Best supportive care	6 (50%)

Abbreviations: Ad, adenocarcinoma; Sq, squamous cell carcinoma.

Table 4 outlines differences in the recurrence location after second surgery, depending on the surgical procedure. Seven out of 12 cases with lobectomy and more than lobectomy relapsed with no stump recurrence. Five out of 22 cases with limited surgery had recurrence. The second surgical procedure was wide‐wedge resection in 15 cases, of which two cases (13%) showed stump recurrence postoperatively.

**TABLE 4:** Difference in recurrence location depending on surgical procedure

Variables	Lobectomy and more than lobectomy (*n* = 12)	Segmentectomy and wide‐wedge resection (*n* = 22)
Intrapulmonary recurrence	3 (25%)	3 (14%)
Mediastinal lymph node	2 (17%)	0 (0%)
Intrapulmonary recurrence and mediastinal lymph node	1 (8%)	0 (0%)
Stump recurrence	0 (0%)	2 (9%)
Bone	1 (8%)	0 (0%)

In group A, 14 patients (41%) died during the course of the study. The cause of death was lung cancer in six cases (43%), other diseases in four cases (29%), ILD‐AE in one case (7%), and unknown in three cases (21%). In group B, eight patients (73%) died during the course of the study. The cause of death was lung cancer in six patients (75%), other diseases in one patient (13%), and ILD‐AE in one patient (13%).

## DISCUSSION

We report the course, prognosis, and surgical results of cases that may be operated on for a second time after surgery for lung cancer with ILD. No previous study has reported the second surgical course in 45 cases of ILD‐complicated lung cancer. Regarding limited surgery and curability of cancer, Ginsberg et al. reported that because of the higher death and locoregional recurrence rates associated with limited resection, lobectomy must still be considered the surgical procedure of choice for patients with peripheral T1 (tumor diameter ≤3 cm) N0 non‐small cell lung cancer.^10^ However, the optimal surgical choice in relation to the timing and extent of resection of the second nodule remains controversial. In the literature, limited surgery is indicated during the second surgery in 44%–82% of cases with or without ILD.^11^, ^12^, ^13^, ^14^, ^15^, ^16^, ^17^ Our limited surgery rate was 65%, which was slightly higher than that of patients without ILD. Optimal management is affected by many factors that should be carefully considered when determining the extent of resection, including age, cardiopulmonary reserve, performance status, and intraoperative frozen section results. However, Fukui et al. indicated that idiopathic interstitial pneumonia increases the risk of underestimating the tumor size of lung cancer on preoperative CT measurements.^18^ We also observed stump recurrence in two of the 34 patients who underwent wide‐wedge resection. Therefore, the extent of the tumor should be evaluated more carefully than usual to maintain an appropriate surgical margin.

Second, the surgical complication rate was 26%, and the 30‐day mortality rate was 2.9%. Second surgery performed in cases with or without ILD was reported to show a complication rate of 13.5%–36.5% and a 30‐day mortality rate of 0–2.5%.^11^, ^12^, ^13^, ^14^, ^15^, ^16^, ^17^ In comparison with other studies, our study showed almost the same frequency of complications and a slightly higher 30‐day mortality rate. Sato et al. reported that surgical intervention for second primary lung cancer may not achieve positive perioperative and long‐term outcomes for patients with a low body mass index (BMI) or a high Carlson comorbidity index (CCI).^19^ Although we did not examine these factors in our cohort because of the low number of cases, we would like to examine the relationship between BMI and CCI and postoperative complications in a larger cohort of cases in the future. A prolonged pulmonary fistula was the most common complication in the present study. All five cases showing this complication underwent the second surgery on the same side as the first operation, indicating the importance of awareness of adhesion detachment and pulmonary fistula closure during surgery. In addition, among the 12 cases in which lobectomy was performed in the second operation, five required HOT. Of these, two cases did not require HOT within 3 months, while three cases required it for the rest of their lives. Although it is not a complication, postoperative respiratory depression should be considered.

The incidence of ILD‐AE after the second operation was 5.9% (2/34 cases), and the mortality rate was 50.0%. Generally, the incidence of ILD‐AE in initial surgery is 9.3% and the mortality rate is 43.9%.^20^ In another study, Sato et al. reported that of 13 patients (23.1%) showed ILD‐AE and all died as a result of ILD‐AE in a study limited to idiopathic pulmonary fibrosis (IPF) during the second operation.^6^ The surgical cases in our hospital did not show a significant incidence of ILD‐AE in the second operation, but both cases in our hospital that showed ILD‐AE involved male patients, a UIP pattern on CT, and lobectomy. Therefore, the JACS risk score was ≥11 points in both cases, which indicated moderate risk. Therefore, in male patients showing UIP on CT who are candidates for a second surgery, limited surgery may be an option, if possible.

The 5‐year overall survival rate after the second surgery for lung cancer with ILD at our hospital was 39.4%. The 5‐year overall survival rate after the second surgery in the literature is 42.0%–94.1%, and the 5‐year survival rate after the second surgery for lung cancer with ILD has been reported to indicate a very poor prognosis.^11^, ^12^, ^13^, ^14^, ^15^, ^16^, ^17^ Furthermore, the group that did not select the second surgery had 1‐ and 3‐year overall survival rates of 60.0 and 20.0%, respectively, which were extremely poor. There are two reasons for the poor prognosis. First, patients who originally had ILD had a poor prognosis at the outset. The median survival time (MST) in cases without AE during the course of IPF was 5.8 years, while that in cases with AE during the course of IPF was 2.9 years. The MST in cases without AE in the course of ILD excluding IPF was not determined, and the corresponding value in cases with AE in the course of ILD excluding IPF was 4.3 years.^21^ Second, the most common cause of death in group A was lung cancer, suggesting that limited resection may not be able to control lung cancer. Two cases of recurrence of the stump of wide‐wedge resection have been observed, and surgery beyond segmentectomy may be desirable in such cases.

This study had several limitations. First, it was a retrospective, single‐institute study in Japan with a small sample size, which may have led to biases in patient selection and the choice of operative approach. Second, although the 5‐year survival probability was provided, the median follow‐up period from the second surgery was less than 3 years, suggesting that the survival results were premature. Third, we should have added the type and severity of ILD, but could not collect the requisite preoperative information. Fourth, there is an opinion that it may not be appropriate to simply compare the survival rates of groups A and B. This is because group B contains patients who may not be able to tolerate surgery, potentially making the performance status of group B and/or organ function worse than group A. Therefore, a large prospective observational study spanning multiple institutions is needed to confirm the complications and prognosis of a second surgery for lung cancer with ILD.

In conclusion, the second surgery group for lung cancer with ILD showed a significantly better prognosis than the nonsurgery group. On the one hand, choosing limited surgery has an advantage of preservation of respiratory function, but a disadvantage of stump recurrence. On the other hand, choosing standard surgery (lobectomy and more than lobectomy) has an advantage of no stump recurrence, but has a disadvantage of decreased respiratory function. The limited surgery rate was 65%, and the ILD‐AE was 5.8%. Only one patient (2.9%) with AE died within 30 days, and we believe that a second operation for lung cancer with ILD is feasible. However, recurrence of the stump after wide‐wedge resection was observed in two of 15 cases (13%), and the surgical procedure should be carefully evaluated in such cases.

## CONFLICT OF INTEREST

The authors declare no conflict of interest.
